# An Assessment of Household Knowledge and Practices during a Cholera Epidemic— Dar es Salaam, Tanzania, 2016

**DOI:** 10.4269/ajtmh.21-0597

**Published:** 2022-09-06

**Authors:** Sae-Rom Chae, Haji Lukupulo, Sunkyung Kim, Tiffany Walker, Colleen Hardy, Ahmed Abade, Loveness J. Urio, Janneth Mghamba, Robert Quick

**Affiliations:** ^1^ Division of Foodborne and Waterborne and Environmental Diseases, Centers for Disease Control and Prevention, Atlanta, Georgia;; ^2^ Tanzania Field Epidemiology and Laboratory Training Program, Dar es Salaam, Tanzania;; ^3^ Division of Global Health Protection, Center for Global Health, Centers for Disease Control and Prevention, Atlanta, Georgia;; ^4^ Ministry of Health, Community Development, Gender, Elderly and Children, United Republic of Tanzania, Dar es Salaam, Tanzania

## Abstract

From August 15, 2015 to March 5, 2016, Tanzania reported 16,521 cholera cases and 251 deaths, with 4,596 cases and 44 deaths in its largest city, Dar es Salaam. To evaluate outbreak response efforts, we conducted a household survey with drinking water testing in the five most affected wards in Dar es Salaam. We interviewed 641 households 6 months after the beginning of the outbreak. Although most respondents knew that cholera causes diarrhea (90%) and would seek care if suspecting cholera (95%), only 45% were aware of the current outbreak in the area and only 5% would use oral rehydration salts (ORS) if ill. Of 200 (31%) respondents reporting no regular water treatment, 46% believed treatment was unnecessary and 18% believed treatment was too expensive. Fecal contamination was found in 45% of water samples and was associated with water availability (*P* = 0.047). Only 11% of samples had detectable free chlorine residual, which was associated with water availability (*P* = 0.025), reported current water treatment (*P* = 0.006), and observed free chlorine product in the household (*P* = 0.015). The provision of accessible, adequately chlorinated water supply, and implementation of social mobilization campaigns advocating household water treatment and use of ORS should be prioritized to address gaps in cholera prevention and treatment activities.

## INTRODUCTION

Cholera, an acute diarrheal disease, is caused by the Gram-negative rod *Vibrio cholerae*, which can swiftly contaminate water systems and food sources, leading to explosive epidemics.^1^ The hallmark of cholera infection is profuse watery diarrhea that can cause rapid dehydration; mortality can be up to 50% if untreated. Globally, between 1.3 and 4.0 million cases of cholera and 21,000 to 143,000 cholera-related deaths are estimated to occur each year.^2^ The African continent experiences a disproportionately high burden of cholera cases and cholera-related deaths.^3^^–^^6^ From 2000 to 2015, 83% of cholera deaths reported by the WHO occurred in sub-Saharan Africa.^7^

During cholera outbreaks, it is essential to ensure proper case management and quickly mobilize cholera prevention resources to communities at risk. Treatment includes prompt rehydration when cholera is suspected, either through oral rehydration or intravenous fluids.^8^ Lifesaving treatment can be started in the home using oral rehydration salts (ORS), particularly when healthcare cannot be accessed urgently. Widespread education regarding the transmission and prevention of cholera is an important intervention.^9^^,^^10^ Source and stored water can be treated cheaply and effectively with sodium or calcium hypochlorite and monitored objectively by measuring free chlorine residual (FCR).^11^ The WHO recommends FCR levels between 0.2 and 0.5 mg/L at the point-of-use during a cholera outbreak.^12^

During the past several years, large cholera outbreaks have affected East African countries including Tanzania, Kenya, Ethiopia, Somalia, and Mozambique.^13^^–^^17^ Cholera is endemic to Tanzania with cases reported every year since 1977.^18^ The largest country-wide outbreak in Tanzania occurred in 1997, resulting in a total of 40,249 cases and 2,231 deaths.^18^ In August 2015, a case of laboratory-confirmed cholera infection in Tanzania was reported in Dar es Salaam, the initial epicenter of the epidemic, where the majority of the population resides in informal settlements; the case count rapidly rose to 3,371 cases and 36 deaths in Dar es Salaam by the end of October 2015, before spreading to other areas of the country over the next few months.^15^ From August 2015 through March 2016, the CDC assisted the Tanzania Ministry of Health, Community Development, Gender, Elderly, and Children with outbreak response activities. These activities included strengthening epidemic surveillance through the development of a central reporting template and mechanism, improving data quality and visualization, and conducting community case investigations. They also addressed case management through trainings and cholera treatment center evaluations; laboratory support through the development of standard operating procedures for cholera rapid diagnostic tests, laboratory assessments, and water quality testing; and national emergency response coordination through the establishment of an emergency operations center. To evaluate the outbreak response efforts on prevention activities in Dar es Salaam, we conducted a household survey to better describe cholera knowledge, cholera prevention, and treatment practices of community members.

## METHODS

In February 2016, a cross-sectional household survey including drinking water testing was conducted in the five wards in Dar es Salaam with the highest cholera case counts during the peak of the epidemic in October 2015. At the time of each interview, the enumerator also asked the respondent to direct him or her to the pharmacy that was the household’s usual source of medications or water treatment products. The enumerator then visited the pharmacy and queried pharmacy staff persons about the price and current availability of ORS and water treatment products.

### Sampling.

On the basis of a conservative assumption that 5% of households would have detectable FCR in their drinking water (two previous evaluations of cholera outbreaks in which chlorine water treatment was promoted found that 17% and 27%, respectively, had detectable FCR^19,20^), statistical power of 90%, confidence limits of ±2%, and a design effect of 1.75, we calculated a target sample size of 768 households. To obtain our study population, we used a two-stage cluster sampling strategy, with the primary sampling unit being a “10-cell,” an existing cluster system in each ward, consisting of 10 to 15 households on average. In total, 192 10-cells were selected using a probability-proportionate-to-size sampling strategy, and four households in each 10-cell were randomly selected for a total of 768 households. We used household lists obtained from community leaders of each ward, and local community leaders accompanied field teams to help locate selected households.

### Data collection.

Open Data Kit software was used for data collection using electronic tablets. Trained enumerators made home visits to interview heads of selected households or someone familiar with household water management practices to assess respondents’ knowledge of cholera transmission, prevention, symptoms, and treatment; determine household hygiene and drinking water practices; and make observations of the household environment. We used WHO definitions for improved (piped, borehole, protected spring or well, bottled, rainwater catchment, community tap/kiosk) and unimproved (surface source, unprotected spring or well, bottled water, tanker truck) water sources.^11^ Safe water storage was defined as water stored in a closed, narrow-mouthed vessel.^21^ Household stored drinking water was tested for FCR using the N, N-diethyl-p-phenylenediamine colorimetric method (Hach Free and Total Chlorine kits; Hach Co., Loveland, CO). One half of household water samples were randomly selected to test for *Escherichia coli* presence, an indicator of fecal contamination, using the Colilert Defined Substrate Technology method (IDEXX.com).

### Statistical analysis.

All data analysis was performed using SAS version 9.4 (SAS Institute, Cary, NC). We described the sociodemographic characteristics of households, caretaker knowledge and attitudes regarding cholera, and household drinking water cost, availability, and practices. Bivariate analyses were conducted to explore household characteristics associated with detectable FCR (≥ 0.2 mg/L) and *E. coli* contamination in drinking water using χ^2^ or Fisher’s exact test as appropriate.

### Informed consent.

The study protocol (MU/PGS/SAEC/Vol. XIV/49) was approved by the institutional review board of Muhimbili University of Health and Allied Sciences located in Dar es Salaam, Tanzania. The survey was determined to be a nonresearch public health response activity by the CDC’s human subjects advisor. Written informed consent was obtained from all survey respondents.

## RESULTS

We completed interviews with respondents from 641 (83%) of 768 targeted households. Of 127 (17%) interviews not completed, 98 (77%) respondents were not home, 12 (9%) had moved, 11 (9%) refused, and six (5%) were ineligible to be enrolled because an individual ≥ 18 years of age was not present.

### Sociodemographic characteristics.

Of 641 respondents, 74% were female with a median age of 35 years (interquartile range [IQR]: 27–46). The median value for household size was four household members (IQR: 3–6) and the median number of children < 5 years old was one child (IQR: 0–1). Most respondents (90%) had completed primary school or higher.

### Cholera knowledge and prevention, and hygiene practices.

Approximately half (45%) of the 641 respondents were aware of the cholera outbreak in the area, and when asked to list information sources, they indicated the following: family member/neighbor/friend (59%), media (51%), community meeting/leader (13%), health worker (7%), community health volunteer (6%), and poster (5%). Diarrhea was identified by 90% of respondents as a principal symptom of cholera, but 8% were unable to name any symptoms. When asked how they would respond to a household member with suspected cholera, 95% of participants said they would go to a health facility, and only 5% reported they would use ORS. Among factors contributing to cholera transmission, poor hygiene was indicated by 82%, followed by drinking bad water (47%) and eating bad or unwashed food (35%). Cholera prevention measures named most frequently by respondents included water treatment (71%), food safety (69%), and handwashing (36%). Fourteen (2%) respondents reported that a suspected cholera case had occurred in the household in the past 6 months; 86% of these reported using ORS to treat ill household member(s) (Table 1).

**Table 1:** Cholera knowledge and household sanitation, Tanzania 2016

	*N*	*n* (%)
Have heard about the cholera outbreak in the area	641	286 (45)
Main symptoms of cholera*	641	
Diarrhea		575 (90)
Vomiting		497 (77)
Fever		55 (8)
Dehydration		38 (6)
Don’t know		49 (8)
What would do for ill household member(s) with cholera*	641	
Seek healthcare		609 (95)
Use sugar-salt solution		45 (7)
Use ORS		33 (5)
Methods of cholera transmission*	641	
Poor hygiene		524 (82)
Drinking bad water		304 (47)
Eating bad or unwashed food		222 (35)
Flies/insects		77 (12)
Don’t know		31 (5)
Cholera prevention measures*	641	
Boil or treat water		454 (71)
Food safety†		441 (69)
Wash hands		228 (36)
Use a latrine/avoid open defecation		45 (7)
Household member became ill with cholera in the past 6 months	641	14 (2)
Used ORS to treat the ill household member(s)	14	12 (86)
Had heard of ORS for cholera		494 (77)
Have ever used ORS in the past	494	267 (42)
Did you buy or received it for free?	267	
Bought		144 (54)
Received for free		112 (42)
Both		5 (2)
Don’t know		6 (2)
Have received ORS for free during the past 6 months	641	41 (6)
Observed ORS in the household	641	15 (2)
Most critical time to wash hands*	641	
Before eating/feeding children		531 (83)
After using the toilet		517 (81)
Before cooking/preparing food		176 (27)
After handling/cleaning children’s stools		61 (10)
Observed handwashing station (with soap and water)	641	326 (51)
Observed latrine	641	597 (93)

ORS = oral rehydration salts.

* Select all that apply.

† Cook food thoroughly, reheat stored food, cover food, washing vegetables/fruit.

Among all respondents, 77% had heard of ORS, and 42% had used it in the past. Of 267 respondents who reported ORS use in the past, 54% reported paying for it, and 42% said they received it at no cost. Overall, 6% of respondents reported having received ORS gratis in the previous 6 months. Enumerators observed ORS in 2% of households at the time of interview.

When asked to name important handwashing times, responses included before eating or feeding children (83%), after using the toilet (81%), before cooking or preparing food (27%), and after handling children’s stools (10%). Functional handwashing stations (having water and soap in place) were observed in 51% of households. Most households (93%) reported having access to a latrine, which was confirmed by enumerators.

### Household water availability and management practices.

Approximately two-thirds of respondents (62%) reported obtaining drinking water from an improved source, including a covered well, borehole, rainwater catchment, piped water, community tap, or kiosk (Table 2). Nearly all survey participants (99%) reported paying for their drinking water, 460 (72%) of whom reported paying an average of USD 0.09 per 20-L bucket, which over the course of a month would cost approximately USD 2.70; at an average income of USD 45 per month,^22^ paying for one bucket of water per day would use approximately 6% of the respondents’ income. More than one-third of households (37%) reported being unable to afford drinking water at least once during the past month, and 68% could not obtain water from their usual source at least once during the same period. The reasons for lack of water included broken or disconnected pipes (33%) and water rationing (29%). Of 441 (69%) respondents who reported regular water treatment, the main treatment methods included boiling (60%) and chlorinating (51%). Of 200 (31%) respondents who reported no regular water treatment, the reasons included the belief that treatment was not necessary (46%), treatment products were too expensive (18%), and habits were difficult to change (18%). Approximately 56% of households reported ever receiving free water treatment or hygiene products; of these respondents, 92% reported using the free items. Household drinking water was most often stored in a bucket (80%); 98% were observed to be covered.

**Table 2:** Household drinking water cost, availability, and practices, Tanzania 2016

	*N* or mean (±SD)	*n* (%)
Improved drinking water source*	638	399 (62)
Pay for drinking water	641	634 (99)
Mean cost of drinking water, USD†		
10-L bucket	0.09 (±0.20)	34 (5)
20-L bucket	0.09 (±0.05)	460 (72)
20-L jerrycan	0.16 (±0.09)	48 (7)
Monthly	9.78 (±3.21)	63 (10)
Unable to afford drinking water ≥ 1 time during past month	633	235 (37)
Usual water source not available ≥ 1 time during past month	636	435 (68)
If yes, reason that drinking water not available‡	435	
Broken pipe		144 (33)
Water rationed/not running		125 (29)
Pipe cut or disconnected		31 (7)
Reported regular drinking water treatment	641	
Yes		441 (69)
Reported water treatment method‡	441	
Boiling		263 (60)
Chlorine products§		225 (51)
Others		54 (12)
No		200 (31)
Reason for no treatment	200	
No need to treat		92 (46)
Habit		35 (18)
Too expensive		35 (18)
Product not available		9 (5)
Do not know how to use the product		9 (5)
Reported current drinking water treatment	641	246 (38)
Have received gratis water treatment or hygiene products	641	357 (56)
Used gratis product	357	327 (92)
Observed drinking water storage container type	641	
Bucket		511 (80)
Jerrycan		48 (7)
Bottles		46 (7)
Others		24 (4)
Observed all storage containers covered	597	587 (98)

* Covered well/borehole/rainwater catchment/piped water/community tap/kiosk.

† Applied 0.0046 USD exchange rate/1 Tanzanian shilling.

‡ Respondents were able to select more than one response.

§ WaterGuard/Aquatabs/bottles of chlorine/Pur (P&G Purifier of Water).

### Household drinking water testing results.

Of 313 (51%) household water samples tested for *E. coli*, 142 (45%) were positive (Table 3). The rate of *E. coli* detection was higher in samples from households that reported their usual water source was not available ≥ 1 time during the past month (103/211, 49%) compared with usual water source always available (36/98, 37%) (*P* = 0.047). It was also higher in samples with undetectable FCR (137/277, 49%) compared with detectable FCR (5/35, 14%) (*P* < 0.001). Overall, FCR was detected in 68 (11%) of 599 water samples tested (Table 4). FCR was more likely to be detected in households where the usual water source was not available ≥ 1 time during the past month (55/410, 13%) compared with always available (13/184, 7%) (*P* = 0.025), in households where respondents reported current water treatment (20/116, 17%) in stored water compared with households with no reported treatment (14/194, 7%) (*P* = 0.006), and in households with an observed chlorine product (47/256, 18%) compared with those with no observed chlorine product (7/83, 7%) (*P* = 0.015).

**Table 3:** Household characteristics associated with *Escherichia coli* contamination in stored water samples (*N* = 313),* Tanzania 2016

Variable	*E. coli* +	*E. coli* –	*P* value
	(*N* = 142, 45%)	(*N* = 171, 55%)	
Education			0.752
Primary school or less	15 (43)	20 (57)	
Primary school or higher	127 (46)	151 (54)	
Age, mean (±SD)	39.5 (±15.2)	39.1 (±14.5)	0.898
Household size, mean (±SD)	5.0 (±2.9)	5.2 (±3.0)	0.300
Water source			0.871
Improved†	90 (46)	107 (54)	
Unimproved	41 (45)	63 (55)	
Usual water source availability			**0.047**
Not available ≥ 1 time during past month	103 (49)	108 (51)	
Always available	36 (37)	62 (63)	
Water storage container			0.078
Improved‡	20 (43)	26 (57)	
Unimproved	122 (46)	145 (54)	
Reported regular water treatment			0.056
Yes	85 (41)	120 (59)	
No	57 (53)	51 (47)	
Reported current water treatment			0.111
Yes	46 (40)	70 (60)	
No	95 (49)	99 (51)	
Free chlorine residual level ≥ 0.2 mg/L			**< 0.001**
Yes	5 (14)	30 (86)	
No	137 (49)	140 (51)	
Observed handwashing station			0.952
Yes	73 (45)	91 (55)	
No	5 (45)	6 (55)	
Observed soap			0.618
Yes	99 (44)	126 (56)	
No	13 (39)	20 (61)	
Observed latrine			0.336
Yes	135 (46)	158 (54)	
No	7 (35)	13 (65)	

* For some characteristics, the counts do not sum to total due to missing values.

† Covered well/borehole/rainwater catchment/piped water/community tap/kiosk.

‡ Defined as a covered narrow-mouthed container such as a jerrycan.

**Table 4:** Household characteristics associated with detectable free chlorine residual (FCR) in stored water samples (*N* = 599),* Tanzania 2016

Variable	FCR	FCR	*P* value
	≥ 0.2 mg/L	< 0.2 mg/L	
	(*n *= 68, 11%)	(*n* = 531, 89%)	
Education			0.759
< Primary school	8 (12)	56 (88)	
≥ Primary school	60 (11)	475 (89)	
Age, mean (±SD)	38.2 (±14.5)	38.5 (±12.4)	0.503
Household size, mean (±SD)	4.9 (±2.8)	5.4 (±2.9)	0.124
Water source			0.066
Improved†	49 (13)	326 (87)	
Unimproved	18 (8)	203 (92)	
Usual water source availability			**0.025**
Not available ≥ 1 time during last month	55 (13)	355 (87)	
Always available	13 (7)	171 (93)	
Water storage container			0.800
Improved‡	4 (9)	42 (91)	
Unimproved	31 (12)	235 (88)	
Reported regular water treatment			0.138
Yes	52 (13)	359 (87)	
No	16 (9)	172 (91)	
Reported current water treatment			**0.006**
Yes	20 (17)	96 (83)	
No	14 (7)	180 (93)	
Reported use of gratis product in the past 6 months			0.903
Yes	49 (16)	263 (84)	
No	4 (15)	23 (85)	
Observed gratis chlorine§			**0.015**
Yes	47 (18)	209 (82)	
No	6 (7)	77 (93)	
Observed handwashing station			0.807
Yes	31 (10)	278 (90)	
No	3 (12)	23 (88)	
Observed soap			0.147
Yes	57 (13)	371 (87)	
No	5 (7)	65 (93)	
Observed latrine			0.577
Yes	62 (11)	494 (89)	
No	6 (14)	37 (86)	

* For some characteristics, the counts do not sum to total due to missing value.

† Covered well/borehole/rainwater catchment/piped water/community tap/kiosk.

‡ Defined as a covered narrow-mouthed container such as a jerrycan.

§ WaterGuard/Aquatabs/bottles of chlorine/Pur (now called P&G Purifier of Water).

### Pharmacy survey.

Of 338 neighborhood pharmacies surveyed, 42% had ORS in stock with an average cost of 0.24 USD per sachet, and 25% had water treatment products available. The most available product, a 10-tablet strip of chlorine tablets, cost 0.25 USD on average, with each tablet treating a 20-L bucket.

## DISCUSSION

This evaluation revealed several conditions that could have contributed to cholera risk. Although knowledge of cholera symptoms and awareness of the importance of seeking care for suspected cholera were considered high, fewer than half (45%) of survey households were aware of the ongoing cholera outbreak. Cholera-aware respondents listed various information sources, but the relatively low awareness of the outbreak highlighted the need for an improved understanding of how communities learn about public health risks and an exploration of other modalities for social mobilization. The percentage of household respondents reporting no regular water treatment was low (31%), and 14% of respondents did not believe their water needed to be treated. Among respondents who did report current water treatment, only 17% had detectable FCR in stored water and 40% had detectable *E. coli* in stored water samples, which suggested that respondents either were falsely reporting treatment (social desirability bias) or treating their water incorrectly. Household drinking water supply was expensive and unreliable, and 45% of samples were contaminated, problematic conditions in a country with recurrent cholera outbreaks. The rate of *E. coli* contamination was higher in the drinking water of households that had to obtain their drinking water from sources other than their usual source during the previous month, highlighting the impact of accessibility on water quality. Interestingly, no association was found between water contamination and improved versus unimproved water source, suggesting either that water sources thought to be improved may have been contaminated or that water may have become contaminated during storage. The uncertain safety of improved water sources in the developing world is well recognized and may underestimate diarrheal disease risk.^23^ Finally, many households used unsafe water storage practices, and only half had handwashing stations.

In this survey, confirmed household water treatment was rare with detectable chlorine in stored water in only 11% of households. Observing a free water treatment product in the home was associated with a detectable level of FCR, which suggested that many respondents either lacked the resources or the motivation to purchase water treatment products and underscored the potential benefit of making household water treatment freely available during an outbreak. Because making large-scale infrastructure improvements during a waterborne disease outbreak is infeasible due to required time and expense, point-of-use water treatment can help protect at-risk communities in the short to medium term.^24^ In rapidly growing cities like Dar es Salaam where the density of informal settlements is high and access to safe water is often limited, point-of-use water treatment should be included in a multifactorial approach to address unsafe water.

Prompt treatment of diarrhea with ORS to prevent severe dehydration is a critically important early intervention in cholera outbreaks and depends on accessibility of ORS in the community.^25^^,^^26^ Our survey found that awareness of ORS and its use in during a cholera outbreak was low. Furthermore, ORS was available in fewer than half of neighborhood pharmacies. Low availability of ORS can lead to increased mortality.^26^ If ORS is not distributed widely for free, it should be readily available at a low price in local kiosks or pharmacies. It is also important to educate and empower communities and caregivers through appropriate and consistent guidance on the purpose, proper preparation, and use of ORS, and expected outcomes.^25^ Social mobilization efforts are integral to assuring ready access and reinforcing early use of ORS at home.^27^^–^^29^

This outbreak highlighted the importance of regional and global partnerships in addressing cholera preparedness and response because outbreaks often occur through cross-border travel or migratory workforces and can continue to affect a region for many years.^13^ During the period of this outbreak, cholera was reported in the Democratic Republic of the Congo (DRC), Kenya, Malawi, Mozambique, Somalia, and Uganda.^30^ In 2015, DRC, Kenya, and Tanzania accounted for 62% of cholera cases reported from Africa. Global weather patterns have been implicated in the geographic distribution of illnesses.^31^^–^^37^ During this period, El Niño, a complex series of weather patterns affecting the equatorial Pacific, brought wetter and warmer weather to East Africa, contributing to a 3-fold increase in cholera incidence in the region.^38^ Regional and global cooperation can facilitate targeting of vulnerable populations for prevention campaigns, including cholera vaccination, which may help limit transmission.

In response to the ongoing problem of cholera across multiple countries in East Africa and beyond, in 2017, the Global Task Force on Cholera Control launched a global, multisectoral strategy for cholera control called “Ending Cholera—A Global Roadmap to 2030.”^39^ The aim of this initiative is to help stimulate efforts to ensure that improved coordination, advocacy, funding, and technical assistance are leveraged toward the goal of reducing global cholera deaths by 90% and supporting at least 20 cholera-endemic countries to completely eliminate local transmission by 2030.^39^ The initiative seeks to target countries in regions such as East Africa that are heavily affected by cholera and to prevent disease recurrence through the improvement of water, sanitation, and hygiene and the provision of other resources, including vaccines, for cholera control. By identifying local areas at elevated risks for cholera (cholera hotspots) and developing and implementing a National Cholera Plan that focuses resources in those areas, health ministries and partner organizations can better prevent, detect, and contain suspected cholera cases and outbreaks in a timely manner and thereby demonstrate national and regional benefits.^40^

This evaluation had several important limitations. First, our data were not representative of Dar es Salaam or Tanzania as we only sampled the five wards most affected during the early stages of the outbreak. Second, because of a lengthy protocol-approval process, data collection was delayed for 3 months and may not have reflected the community’s knowledge, practices, and conditions during the early stages of the epidemic. On the other hand, our data may serve as an assessment of the impact of early outbreak response-related interventions on the affected population. Third, our survey may have been subject to information bias because we were unable to capture individuals who were working during the daytime hours of the survey or were otherwise difficult to locate. Fourth, because the number of households included in the study was smaller than the target of 768, we had limited power for the household drinking water analysis. For instance, of 313 households that were tested for *E. coli*, only about half (*N* = 175) had data available for the observed hand-washing station in households because many stations were either located too far from the house to visit or were locked. Finally, because of considerable stigma and shame surrounding cholera in Dar es Salaam at the time of the survey, some households may not have been willing to be interviewed or to acknowledge their direct experience with cholera.

Tanzania continues to experience significant risk of cholera due to its low coverage of improved and reliable water, sanitation, and hygiene infrastructure and challenges in developing and implementing a comprehensive National Cholera Plan.^41^ Our evaluation revealed that communities still had not received many key messages and lacked prevention and treatment resources despite ongoing response efforts. Targeted social mobilization efforts are an essential element of National Cholera Plans that includes the development and communication of key messages for cholera prevention, including the importance of treatment and safe storage of drinking water, and can contribute to necessary community behavior change.^42^
